# Microbiota transplantation: concept, methodology and strategy for its modernization

**DOI:** 10.1007/s13238-018-0541-8

**Published:** 2018-04-24

**Authors:** Faming Zhang, Bota Cui, Xingxiang He, Yuqiang Nie, Kaichun Wu, Daiming Fan, FMT-standardization Study Group, Baisui Feng, Dongfeng Chen, Jianlin Ren, Mingming Deng, Ning Li, Pengyuan Zheng, Qing Cao, Shaoqi Yang, Xingxiang He, Yu Liu, Yuqiang Nie, Yongjian Zhou, Daiming Fan, Kaichun Wu, Yongzhan Nie, Guozhong Ji, Pan Li, Bota Cui, Faming Zhang

**Affiliations:** 1 Medical Center for Digestive Diseases, The Second Affiliated Hospital of Nanjing Medical University, Nanjing, 210011 China; 2 Key Lab of Holistic Integrative Enterology, Nanjing Medical University, Nanjing, 211166 China; 3 Division of Microbiotherapy, Sir Run Run Shaw Hospital, Nanjing Medical University, Nanjing, 211166 China; 4 Department of Gastroenterology, The First Affiliated Hospital of Guangdong Pharmaceutical University, Guangzhou, 510080 China; 5 Department of Gastroenterology, Guangzhou Digestive Disease Center, Guangzhou First People’s Hospital, Guangzhou Medical University, Guangzhou, 510180 China; 6 State Key Laboratory of Cancer Biology & Xijing Hospital of Digestive Diseases, Fourth Military Medical University, Xi’an, 710032 China; 7 National Clinical Research Center for Digestive Diseases, Xi’an, 710032 China

**Keywords:** selective microbiota transplantation, microbiome, bacteria, *Clostridium difficile*, inflammatory bowel disease, step-up fecal microbiota transplantation, perspectives

## Abstract

Fecal microbiota transplantation (FMT) has become a research focus of biomedicine and clinical medicine in recent years. The clinical response from FMT for different diseases provided evidence for microbiota-host interactions associated with various disorders, including *Clostridium difficile* infection, inflammatory bowel disease, diabetes mellitus, cancer, liver cirrhosis, gut-brain disease and others. To discuss the experiences of using microbes to treat human diseases from ancient China to current era should be important in moving standardized FMT forward and achieving a better future. Here, we review the changing concept of microbiota transplantation from FMT to selective microbiota transplantation, methodology development of FMT and step-up FMT strategy based on literature and state experts’ perspectives.

## INTRODUCTION

Fecal microbiota transplantation (FMT), a concrete evidence proving the role of microbiota in many diseases, has recently become a research focus in biomedicine and clinical medicine (Khoruts and Sadowsky 2016; Lynch and Pedersen 2016; Vindigni and Surawicz 2017). It has been approved as a standard therapy for recurrent *Clostridium difficile* infection (CDI) by official guidelines (Surawicz et al., 2013). Besides, trials have revealed its potential role in dealing with refractory ulcerative colitis (Cui et al., 2015b; Kellermayer et al., 2015; Moayyedi et al., 2015; Rossen et al., 2015), Crohn’s disease (Zhang et al., 2013; Cui et al., 2015a; Shimizu et al., 2016; Bak et al., 2017; He et al., 2017a, b), constipation (Tian et al., 2016), irritable bowel disease (IBS) (Johnsen et al., 2018), liver disease (Kao et al., 2016; Bajaj et al., 2017; Philips et al., 2017; Ren et al., 2017), blood disease (Kakihana et al., 2016; Spindelboeck et al., 2017), autism (Kang et al., 2017) and epilepsy (He et al., 2017a). According to the data on clinicaltrials.gov., more than 200 trials have been or are being conducted by the end of March 2018, most in the past two years. These trials are mainly focused on indications beyond CDI, such as inflammatory bowel disease (IBD). Only a few are designed to analyze the methodology, clinical decision, safety and cost-efficacy of FMT. A multidisciplinary survey by a group of international clinicians (Stallmach et al., 2018) showed the different perceptions on the use of FMT in IBD patients. More questions on indications beyond CDI and IBD remain unanswered. We have stepped on the long journey of standardizing FMT. In this review, we will discuss the changing concept, methodology development, management strategy of microbiota transplantation from literature and propose our perspectives.

## EXPERIENCES OF USING FECAL MICROBIOTA IN HISTORY

FMT is a breakthrough not in technological or theoretical research, but in medical recognition. The sequencing of microbiota, bioinformatics technology and holistic understanding on microbiome provided new intuitionistic evidence for indicating the complex community-intrinsic properties (Dubilier et al., 2015; Madsen et al., 2017). However, in-depth researches on microbiota are very limited. In a recent study, the Yin-Yang theory (Yin and Yang) originating from traditional Chinese medicine has been proposed to explain the complex ecosystem of gut microbiota (Gibson et al., 2014; Wu et al., 2016; Starkel et al., 2018). According to this theory, the Yin-Yang balance is established between the harmful and beneficial microbial cells in the intestine, a process in which two contradictory forces complement and give rise to each other (Cui and Zhang 2018). Once this balance collapses, pathogens will invade and diseases will occur.

The history of using the stool of the healthy to treat human diseases can date back to the fourth century in China (Zhang et al., 2012b). In Dong Jin Dynasty (AD 300–400 years), Hong Ge, who composed the emergency medicine book *Zhou Hou Bei Ji Fang* (Ge 2000), described the details of using fecal suspension for serious disorders, including food poisoning, “Wen Bing” (febrile disease) and “Shang Han” (typhoid fever). Therefore, to our knowledge, this book is the first record of using human feces to treat human diseases (Zhang et al., 2012b). The ancient medical history of FMT was confirmed by the following criteria: (1) the delivered materials are taken from human fecal matter; (2) the administration route is the digestive tract; (3) the efficacy is caused by microbiota from the fresh fecal water or fermented fecal matter according to the modern medicine; (4) the recorded prescription, methods, indications and efficacy in ancient literature are clear enough to be identified. Ge clearly recorded this medical use in his book, which suggesting that FMT might have been used in folk medicine long before his era. Expressions like “Wen Bing” and “Shang Han” were then used to classify diseases. “Wen Bing” and “Shang Han” with diarrhea, bloody and purulent stool were classified as refractory conditions in *Zhou Hou Bei Ji Fang* (Li 2011). According to *Ben Cao Gang Mu* authored by Shizhen Li in 1578, fresh or fermented human fecal water could be used for “Wen Bing” with super-high fever, poisoning, abscess, phlegm, stagnated food, or “internal heat” (Li 2011). The book *Chong Ding Tong Su Shang Han Lun* (Revised Common Discussion of Cold Pathogenic Febrile Disease) (Xu 2011) documented that fecal solution or children’s feces could be used to treat “Shang Han” with serious diarrhea. This fecal therapy has also been used for refractory diseases by some elder-generation physicians in recent decades in China. The long tradition of using fecal therapy in China might contribute the high acceptance of FMT by Chinese physicians according to Yang’s survey in 2014 (Ren et al., 2016). In 1958, Eiseman et al. reported in English the use of fecal enemas for patients with severe pseudomembranous colitis (Eiseman et al., 1958). In 2013, FMT was for the first time coined into the treatment guidelines on recurrent CDI (Surawicz et al., 2013). Accumulating evidence about FMT opens a new window into the treatment of microbiota associated diseases (Table 1).

**Table 1:** The important events using fecal microbiota transplantation in history

History of fecal microbiota transplantation (FMT)	Significance of the events
**The fourth century** (Ge 2000; Zhang et al., 2012b) Ge Hong, a Chinese doctor described the rescue for serious food poisoning, fever, diarrhea by drinking fecal water or fermented fecal matter in the book “Zhou Hou Bei Ji Fang”	The clear methods and indications supported the wide practice in the following 1,700 years in China
**1590** (Li 2011; Zhang et al., 2012b) Li Shizhen, a Chinese doctor described more than 20 conditions effectively treated by fecal water or fermented fecal matter in the book “Ben Cao Gang Mu”	The most complete record in medical use of fecal matter in traditional human medicine
**1958** (Eiseman et al., 1958) Eiseman et al. from America successful treatment for pseudomenbraneous colitis using fecal water by enema	The first report of FMT in English literature
**1989** (Bennet and Brinkman 1989; Borody et al., 1989) Bennet and Brinkman from America reported success by fecal water enema for Bennet’s ulcerative colitis; Borody et al. in Australia reported fecal water enema in 55 patients with constipation, IBS and IBD	They broadened the use of FMT in intestinal diseases
**2012** (Hamilton et al., 2012) Hamilton et al. from America reported the method and efficacy of using frozen fecal microbiota for *C*. *difficile* infection	The improved methodology for storing fecal microbiota
**2012** (Vrieze et al., 2012) Vrieze et al. from the Netherland reported the FMT in metabolic syndrome	The evidence to use FMT in changing insulin sensitivity
**2013** (Surawicz et al., 2013) FMT was recommended for the treatment of the third occurrence of *C*. *difficle* infection in guideline	FMT was not folk remedy in America since 2013
**2013** (Zhang et al., 2013) Zhang et al. from China reported the FMT through mid-gut in severe fistulizing Crohn’s disease with abdominal inflammatory mass	The evidence to use FMT from enteral disease to severe infection within abdominal cavity
**2015** (Cui et al., 2015b) Cui et al. from China reported the automatic purification of microbiota from stool and the step-up FMT strategy	The modernized process of FMT and improved recognition on FMT strategy
**2016** (Kao et al., 2016) Kao et al. in Canada reported the FMT for hepatic encephalopathy	Provides an evidence to use FMT for a liver disease
**2016** (Kakihana et al., 2016) Fujioka et al. from Japan reported the FMT for acute graft-versus-host disease (GvHD) of the gut	The new option for anti-GvHD by FMT
**2017** (He et al., 2017a) He et al. from China reported the FMT for epilepsy	Provides an evidence to use FMT for a neurological disease

## THE CHANGE IN USING MICROBES TO TREAT DISEASES FROM MICROBE TO MICROBIOTA

**Figure 1:**
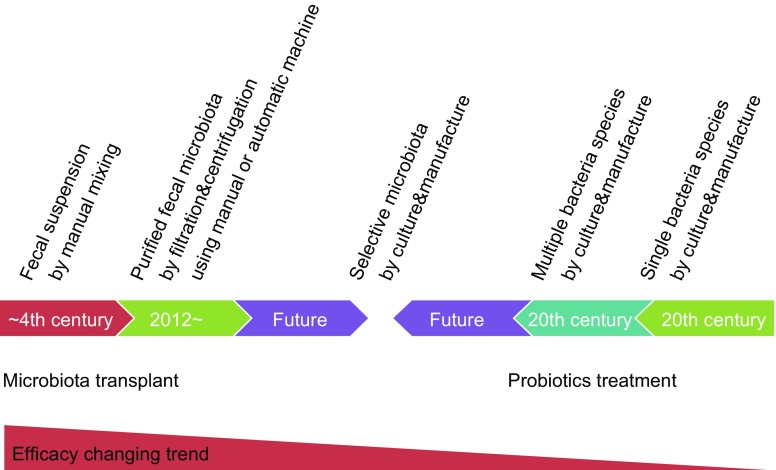
The changing of using microbial cells to treat human diseases

Microbiologists have isolated many bacteria and used them as probiotics in the last century. The use of bacteria as probiotics has a long history (Brodmann et al., 2017). However, the clinical benefit of using a single species of bacteria is limited (Miller et al., 2016). A single microbe has a weak ability in the prevention and treatment of human diseases, though researches have proven its efficacy in standard animal models (Gibson et al., 2017). The different clinical response between using probiotics and microbiota is the core significance of microbiota in human health. Therefore, new microbes and multiple species are being explored for remodeling gut microbiota. Andrews and Borody for the first time reported that a mixture of 18 strains of probiotics could relieve chronic constipation and IBS (Andrews and Borody 1993). The microbial ecosystem therapeutic (MET-1, or “RePOOPulate”) in pilot trials showed an attractive response (Petrof et al., 2013; Martz et al., 2017). The concept of using microbes to treat disease is changing from single species to microbiota (Fig. 1) based on the clinical findings. The use of the whole microbiota is FMT, but FMT does have limitations, such as variable methods, esthetics consideration and safety concerns. The most obvious methodology difference of FMT between the current era and ancient era is based on the centrifugation, cryopreservation and automatic purification. How to preserve FMT materials remains a methodological challenge (Hale et al., 2016). Freshly collected feces can be immediately used, but not stored. The frozen microbiota is prepared with modern cryopreservation. The efficacy of fresh and frozen fecal materials has no significantly difference (Tang et al., 2017), but this finding is mainly from some trials on CDI treatment (Hamilton et al., 2012; Lee et al., 2016). In a double-blind study (Jiang et al., 2017), 72 patients with CDI were randomized to receive fresh, frozen or lyophilized FMT product via colonoscopy. The cure rate (25/25, 100%) was the highest in the group receiving fresh product, lowest (16/23, 78%) in the lyophilized group and intermediate (20/24) in the frozen group. The frozen materials lost a large proportion of bacteria and decreased its efficacy in treating IBD (Cui et al., 2015a, b). This is the reason why the FMT using fresh materials is recommended in our center (He et al., 2017a, b; Zhang et al., 2017). The high clinical efficacy of fresh FMT is also observed in other centers (Uygun et al., 2017). Therefore, the clinical options should refer to the preservation methods of fecal microbiota when necessary.

## FROM FMT TO SELECTIVE MICROBIOTA TRANSPLANTATION

It sounds an easy job for patients to understand the transplantation of microbiota into gut. The recent survey (Xu et al., 2016) on patients’ attitude showed that 56.19% of 105 patients with Crohn’s disease presented satisfactory clinical efficacy and 74.29% were willing to receive the second FMT. Additionally, 89.52% (94/105) showed their willingness to recommend FMT to other patients. This study for the first time demonstrated that patients with Crohn’s disease are willing to receive FMT due to its efficacy. Also, several cost-efficacy analyses of FMT for CDI in America (Konijeti et al., 2014; Varier et al., 2015), Canada (Waye et al., 2016), Australia (Merlo et al., 2016) and France (Baro et al., 2017) and IBD in China (Zhang et al., 2017) have demonstrated its significant advantage in reducing medical and social costs.

However, it is not easy for patients to accept the microbiota transplantation into organs beyond gastrointestinal tract. The accumulating evidences have shown a potential role of microbiota in extra-intestinal sites, such as vagina (Klatt et al., 2017), sinus (Schwartz et al., 2016), urinary tract (Tariq et al., 2017) and skin (Chu et al., 2017a, b). Therefore, the research in the future should focus on the specific use of microbiota in different organs (Hoffmann et al., 2017a, b), a strategy called selective microbiota transplantation (SMT). When SMT is used in gut for simulation of FMT, it can be named as mini-FMT. The possible applications of microbiota transplantation in human disease are shown in the Table 2.

**Table 2:** Possible usage of microbiota transplantation in human diseases

Types	Sites	Microbiota profile	Suitable dosage forms	Possible management in policy
FMT	Microbiota to gut	Whole	Pill, suspension	Medical technology or drug
SMT/mini-FMT	Microbiota to gut	Selected	Pill, powder, suspension	Medical technology or drug
SMT	Microbiota to sinus	Selected	Powder, spray, suspension	Medical technology, drug or heath products
SMT	Microbiota to vagina	Selected	Pill, powder, suspension	Medical technology or drug
SMT	Vaginal microbiota to skin	Selected/whole	Powder, spray, suspension	Medical technology, drug or heath products

Therefore, microbiota transplantation includes the whole profile of microbiota transplantation (e.g., FMT and vaginal microbiota transplantation) and the SMT for the stimulation of the whole profile of microbiota (e.g., the intermediate composition of bacteria between traditional probiotics and whole profile of microbiota). SMT should be promising in precision medicine.

**Figure 2:**
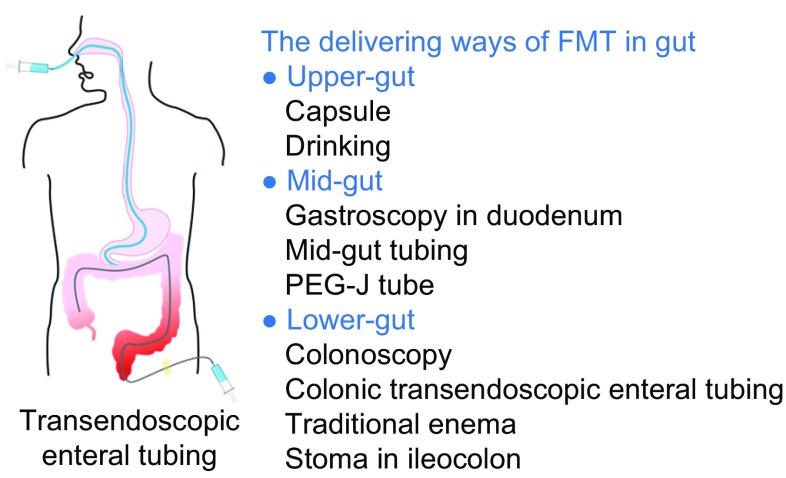
The delivering ways of microbiota transplantation in gastrointestinal tract

## DELIVERING WAYS FOR MICROBIOTA TRANSPLANT

Delivering ways for microbiota transplantation (Fig. 2) include the upper gut, mid-gut and lower gut (Peng et al., 2016). Oral intake of microbiota capsules is a mean of delivery through the upper gut (Youngster et al., 2014; Lee et al., 2016). Selected microbiota can be made into suspension or powder  forms. The microbiota suspension can be infused into the small intestine beyond the second duodenal segment through endoscopy (Zhang et al., 2012a), nasojejunal tube (Cui et al., 2015a, b), mid-gut transendoscopic enteral tubing (TET) (Long et al., 2018), small intestine stoma or percutaneous endoscopic gastro-jejunostomy (PEG-J) (Ni et al., 2016; Peng et al., 2016). The TET through mid-gut is a novel, convenient and safe procedure for microbiota transplantation that results in a high degree of patients’ satisfaction (Long et al., 2018). Fecal microbiota can be also delivered to the lower gut through colonoscopy, enema, distal ileum stoma, colostomy and colonic TET (Peng et al., 2016). Among them, colonic TET does not affect the patients’ life quality. And 98.1% of cases (53/54) were satisfied with FMT delivered through colonic TET. The colonic TET is recommended for patients who need frequent FMTs or FMT combined with other medications. For each delivering way, the aesthetic factors, psychology and privacy should be considered.

Two deaths have been reported associated with FMT delivering procedures though mid-gut (Baxter et al., 2015; Goldenberg et al., 2018). The two deaths had aspiration and died of pneumonia after mid-gut FMT. This complication can be avoided by the following clinical work-flow for mid-gut delivering: (1) Fasting for at least 4 h before FMT, and increasing gastrointestinal motility using metoclopramide 10 mg by i.m. 1 h before FMT; (2) Nasojejunal tube should be inserted if anaesthesia is not suitable for endoscopy or the patient’s condition is critical; (3) Keeping the patient in a sitting position for mid-gut delivery tube if the patient’s condition permits and has the inserted tube; (4) Keeping the patient in reverse Trendelenburg and incline position (>30°) during endoscopy under anaesthesia, whereas a horizontal position should be avoided; (5) Ensuring patient’s psychological well-being, through patient informed consent, detailed explanation on FMT and monitored anesthesia care during the procedure. It is advisable that patients do not witness the infusion during the procedure.

## LABORATORY PREPARATION OF FECAL MICROBIOTA

The laboratory preparation, critical for a successful FMT, can be classified into “rough filtration (RF)”, “filtration plus centrifugation (FPC)”, “microfiltration plus centrifugation (MPC)” (He et al., 2017a, b). For example, the method in Hamilton’s report in 2012 could be called as FPC (Hamilton et al., 2012), and the automatic method using purification system based on GenFMTer (FMT Medical, Nanjing, China) as MPC (He et al., 2017a, b) that can improve the standardization of the laboratory processes and avoid the technicians’ exposure to fecal matter.

A recent study (Chu et al., 2017a, b) reported that some preparation methods can damage the content of living fecal microbes and the suitability of clinical fecal materials. *Faecalibacterium prausnitzii—*an anti-inflammatory commensal bacterium linked to inflammatory bowel disease*—*decreases once exposed to oxygen. Actually, those manual preparations are generally finished within six hours (Cammarota et al., 2017)—“six-hour FMT protocol”. With an automatic purification system and close cooperation between the laboratory technicians and clinicians, we have shortened the time “from defecation to infusion” or “from defecation to freezing” to one hour (Cui et al., 2016; Cammarota et al., 2017). This “one-hour FMT protocol” greatly improves the clinical response and cost-effectiveness of FMT for IBD patients according to the reports from China and others countries (Cui et al., 2015a, b; He et al., 2017a, b; Uygun et al., 2017; Zhang et al., 2017). Practitioners can easily master this “one-hour FMT protocol” using automatic purification system based on GenFMTer.

## SAFETY AND QUALITY CONTROL OF BENEFICIAL MICROBIOTA

FMT related adverse events should be prevented in specific cases, especially those with poor immune status (Fischer et al., 2016a, b). During middle gut FMT, inappropriate techniques and procedures of infusing microbiota into the small bowel may cause adverse events, including nausea, vomiting and aspiration (Gweon et al., 2016; Furuya-Kanamori et al., 2017). With X-ray fluoroscopy or other non-invasive techniques, a nasojejunal tube should be inserted into the patient’s intestine when the patient is at high risk of aspiration under anesthesia. Diarrhea and fever may occur within three hours after FMT, but this generally does not require use of medications (Cui et al., 2015a, b).

The safety in a long term should be considered, though evidence is solid so far. A woman developed new-onset obesity after receiving stool from a healthy but overweight donor (Alang and Kelly 2015). The potential cardiometabolic diseases, autoimmune diseases and neurological diseases have been discussed (Brandt et al., 2012; Kelly et al. 2015). Wong et al. (2017) reported that the fecal microbiota from patients with colon cancer promoted tumorigenesis in germ-free and carcinogenic mice. Therefore, the strict donor screening should be conducted to prevent the disease transmission through FMT. The American Gastroenterological Association (AGA) is using data from national registry to work out a long term program that evaluates the risks and benefits of FMT for CDI (Kelly et al., 2017). The FmtBank (www.fmtbank.org) is carrying out a non-profit FMT research plan in China (China Microbiota Transplantation System) (Cui and Zhang 2018), covering the treatment decision, therapy, evaluation and safety in a short term and a ten-year follow-up.

## STRATEGY OF USING FMT: STEP-UP FMT STRATEGY

The primary and secondary cure rate of fresh FMT for CDI is 91% and 98%, respectively (Brandt et al., 2012). In an RCT study, these two rates become 81% and 94% of resolution of CDI after the first FMT and repeat FMTs (van Nood et al., 2013). The frozen FMT has been proven to have similar efficacy for treating CDI (Hamilton et al., 2012; Lee et al., 2016). An analysis based on 80 patients demonstrates that FMT is safe in immunocompromised cases (Kelly et al., 2014). A multivariable analysis (Fischer et al., 2016a, b) demonstrates that predictors of early FMT failure include severe or severe/complicated CDI, inpatient status during FMT, and previous CDI-related hospitalization.

Severe and severe/complicated CDI can result in intensive care unit admission, sepsis, toxic megacolon and even death. For them, colectomy is the standard treating strategy but it has a mortality of about 50%. Fischer et al. reported (Fischer et al., 2015) that 29 CDI patients at high risk of colectomy underwent FMT plus vancomycin for severe complicated CDI. Single FMT was performed in 62%, and multiple FMTs in 38% of patients (two FMTs in 31% and three FMTs in 7% of patients). FMT and continued vancomycin in selected patients increased the cure rate.

FMT can be performed for CDI with good efficacy, but the evidence for IBD and other diseases is more controversial. Paramsothy et al. (2017) reported that FMT with intensive doses and multiple donors induced clinical remission and endoscopic improvement in active ulcerative colitis and this treatment was associated with distinct microbial changes. He et al. recently reported that the second fresh FMT was an effective and safe treatment to maintain clinical response in Crohn’s disease three months after the first FMT (He et al., 2017a, b). “One-hour FMT protocol” was reformed FMT in this study. All patients were suggested to receive an initial FMT followed by repeated FMTs every three months. Then 68.0% (17/25) and 52.0% (13/25) of patients achieved clinical response and clinical remission at three months post the initial FMT, respectively. The proportion of patients at 6 months, 12 months and 18 months achieving sustained clinical remission after sequential FMTs was 48.0% (12/25), 32.0% (8/25) and 22.7% (5/22), respectively; 9.5% (2/21) achieved radiological healing and 71.4% (15/21) achieved radiological improvement. The conclusion could be made that multiple fresh FMTs induces and maintains clinical remission in Crohn’s disease complicated with abdominal inflammatory mass.

In a recent pilot study (Cui et al., 2015a, b), 8 of 14 (57.1%) patients achieved clinical improvement and were able to stop steroids use following step-up FMT. Among the 8 responders, 5 (35.7%) received one FMT therapy, 1 (7.1%) received two FMTs, and 2 (14.2%) received two FMTs plus a scheduled course of steroids; 6 patients (42.9%) failed to meet the clinical improvement criteria and maintained steroid dependence, though 3 patients experienced transient or partial improvement. No severe adverse events occurred during treatment and follow-up. Step-up FMT strategy as a holistic integrative concept (Fan 2017) focuses on the specificity of patients with steroid-dependent IBD (Cui et al., 2015a, b, 2016). We further refined the step-up FMT strategy for more than 3,000 cases with CDI, IBD and other disorders.

**Figure 3:**
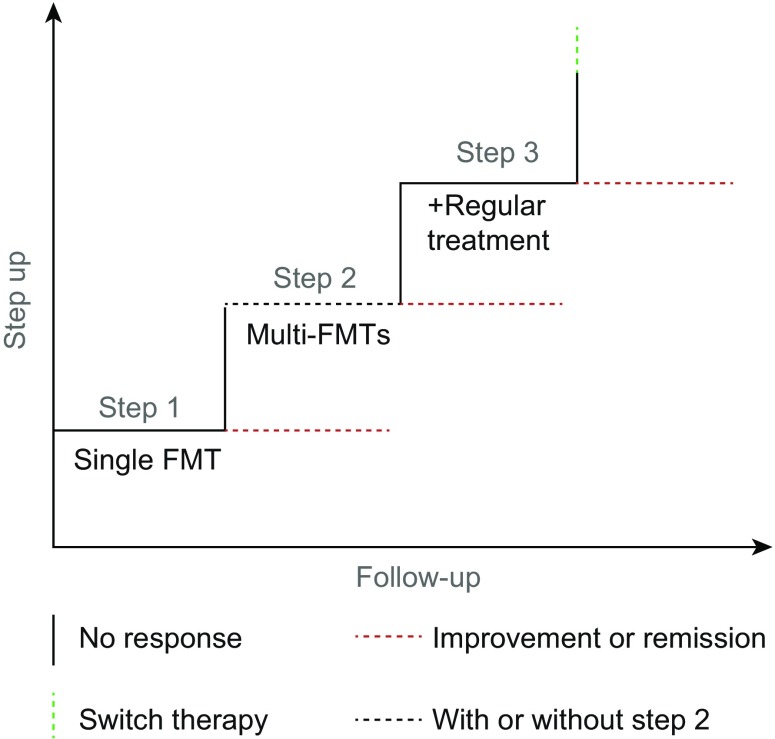
The step-up FMT strategy

As shown in Fig. 3, the step-up FMT strategy consists of three parts: step 1 refers to single FMT; step 2 refers to multi-FMTs (≥2); step 3 refers to the FMT combined with regular medication (such as steroids, cyclosporine, anti-TNF-α antibody, total enteral nutrition) after the failure of step 1 or step 2. The efficacy of each step is enhanced by the measures in the next step. Medication is used at step 3 because the reconstructed gut microbiota may alter the host’s immune status, intestinal barrier, and the sensitivity to regular medicine. This step-up FMT strategy is best indicated for patients with refractory IBD and immune-related diseases (Cui et al., 2015a, b, 2016), severe or complicated CDI (Fischer et al., 2015), especially when patients do not respond to regular medications.

Increasing evidence highlighted the necessity to formulate a treatment ladder with step 1, step 2 and step 3 (Fig. 3). Most commonly, step 1 involves a single FMT for treating CDI or refractory intestinal infection (Surawicz et al., 2013; Wei et al., 2016). The step 2 refers to multi-FMTs and is commonly indicated to treat patients with IBD (Cui et al., 2015a, b, 2016) and partially refractory CDI (Lee et al., 2014). Recently, Suskind et al. reported nine cases of Crohn’s disease undergoing multiple FMTs through the nasojejunal tube (Suskind et al., 2015). 77.8% (7/9) achieved clinical remission two weeks later, and five patients stopped additional medication 12 weeks later. Seth et al. (2016) reported a case of ulcerative colitis in India using multiple FMTs who maintained both clinical and endoscopic remission for more than eight months. Liu et al. (2017) reported multiple FMTs induced remission in 17 of 19 infants with infantile allergic colitis.

**Figure 4:**
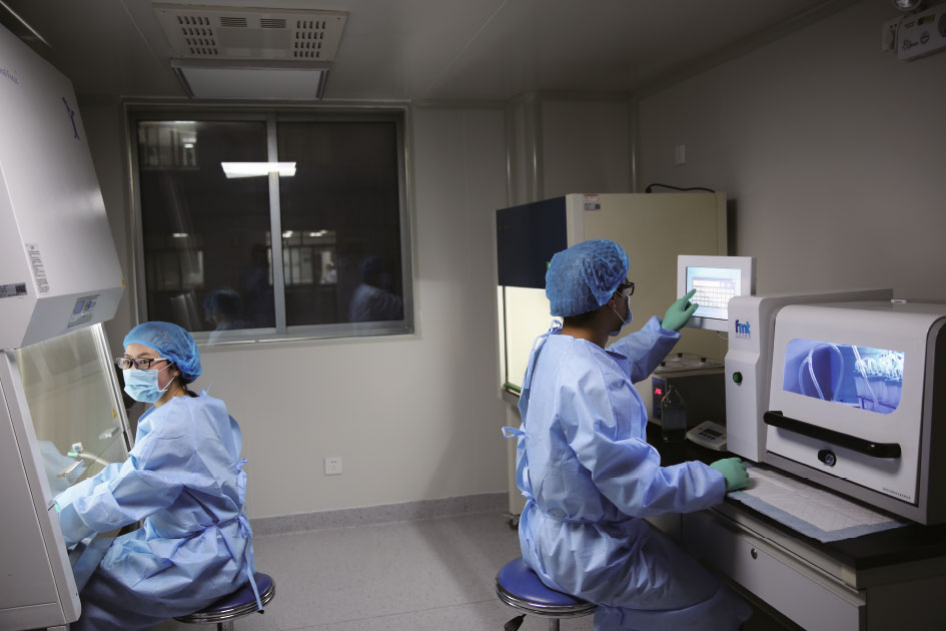
The GMP laboratory for preparation of fecal microbiota

The strategy using steroid after multiple FMTs (e.g., step 3) for steroid-dependent IBD patients has gained more support from clinical researches. Shimizu et al. (2016) reported that one child with ulcerative colitis, who was dependent on high steroid doses and did not respond to anti-tumor necrosis factor alpha (anti-TNF-α) treatment, achieved clinical remission and low-dose steroid dependence after multiple FMTs. Its efficacy could also be found in steroid-ineligible severe alcoholic hepatitis (Philips et al., 2017) and steroid-resistant acute GvHD (Kakihana et al., 2016). These reports, including Fisher’s report on severe CDI (Fischer et al., 2015), have given us a lens to see the potential of this new therapy in treating more microbiota-related diseases, especially those refractory conditions.

## NOVEL STRATEGY OF USING MICROBIOTA TO TREAT CANCER

The increasing researches on immunotherapy, chemical therapy and radiation therapy after remodeling microbiota were reported as promising strategy to treat cancer in recent years. A new research found that gut microbiome improved efficacy of PD-1-based immunotherapy against epithelial tumors (Routy et al., 2018), implying that FMT can be used to fight against cancer. A significant association has been observed between commensal microbial composition and clinical response of anti-PD-L1 therapy in metastatic melanoma patients (Matson et al., 2018). Responders to anti-PD-L1 therapy contain abundant bacterial species like *Bifidobacterium longum*, *Collinsella aerofaciens* and *Enterococcus faecium*. Anti-PD-L1 therapy in germ-free mice models with fecal materials from responding patients showed stronger tumor control, augmented T cell responses, and better efficacy. Another study (Gopalakrishnan et al., 2018) also demonstrated enhanced systemic immunity and favorable gut microbiome profile in patients showing good response to PD-1 immunotherapy, as well as in germ-free mice receiving fecal transplants from responding patients. Food and Drug Administration (FDA) has approved indications of anti-PD-1 and anti-PD-L1 therapies in cancer based on some registered trials (Gong et al., 2018). Another study also showed that gut microbiota could affect anti-cancer response to immunotherapy with CTLA-4 (Vetizou et al., 2015). The bioinformatic and functional studies demonstrated that *Fusobacterium nucleatum* enhanced the resistance of colorectal cancer to chemotherapy (Yu et al., 2017). *Enterococcus hirae* and *Barnesiella intestinihominis* can strengthen Cyclophosphamide-induced therapeutic immunomodulatory effects in cancer (Daillere et al., 2016). Microbiota could be modified in clinical practice to improve its efficacy and reduce the toxic burden of these compounds (Alexander et al., 2017). The effect of radiation on the gut microbiota, and the clinical implications of a modified microbial balance after radiotherapy are now beginning to emerge (Ferreira et al., 2014). FMT could mitigate radiation-induced toxicity and increase the survival rate of irradiated mice. In this process, the peripheral white blood cell counts, gastrointestinal tract function and intestinal epithelial integrity were improved (Cui et al., 2017). These human, animal and in vitro studies suggest that step-up FMT may be a promising strategy in modulating cancer progression and drug response. In the new era of using selected microbiota for transplantation, the strategy of using SMT for cancer treatment should be same as step-up FMT.

## THE UPDATED SAFETY AND MONITORING OF FMT

Serious adverse events can be caused by contaminated microbes in the donor stool. Hence, the laboratory preparation of FMT materials should meet the requirements of that the Good Manufacturing Practice (GMP) set for pharmaceutical companies to manufacture oral medications (Fig. 4). The unqualified human, animals, or biological samples must be excluded. Stools from donors known to the physicians or patients still need consistent screening to rule out infectious pathogens. Thus, a rapid, accurate and convenient fecal pathogen detection method is essential (Hoffmann et al., 2017a, b). To achieve a better traceability, donor fecal samples should be stored in deep cryopreservation for at least two years (Cui et al., 2016). Though FMT-related short term adverse events are in low incidence and mild (Wang et al., 2016), the long-term evaluation on FMT safety should be performed. This is the significance of national register for 10 years of evaluation of FMT in America (Kelly et al., 2017) and China Microbiota Transplantation System. In addition, government authorities must prioritize development of appropriate and effective regulation of FMT to safeguard patients and donors, promote related research and avoid abuse of the treatment (Ma et al., 2017).

## CONCLUSIONS

In conclusion, the main changing concept of using microbial cells is to use microbiota as a holistic integrity. Emerging evidence on FMT revolutionizes our understanding on the mechanism and treatment of microbiota-related diseases. It is time to end the very crude stool transplant in humans. It is time to develop standardized FMT into a mainstream therapeutic option to bring benefits to more patients. The conditions of FMT will cover more diseases beyond recurrent CDI. SMT in specific organ will be a promising therapeutic choice in the near future. The strategy of using microbiota should attract more attention and become widely acceptable in biomedicine research and clinical decision-making.
